# All together now: Analogies between chimera state collapses and epileptic seizures

**DOI:** 10.1038/srep23000

**Published:** 2016-03-09

**Authors:** Ralph G. Andrzejak, Christian Rummel, Florian Mormann, Kaspar Schindler

**Affiliations:** 1 Universitat Pompeu Fabra, Department of Information and Communication Technologies, Barcelona, Spain; 2 Support Center for Advanced Neuroimaging, University Institute for Diagnostic and Interventional Neuroradiology, Inselspital, Bern University Hospital, University of Bern, Bern, Switzerland; 3 Department of Epileptology, University of Bonn, Bonn, Germany; 4 Schlaf-Wach-Epilepsie-Zentrum (SWEZ), Department of Neurology, Inselspital, Bern University Hospital, University of Bern, Bern, Switzerland

## Abstract

Conceptually and structurally simple mathematical models of coupled oscillator networks can show a rich variety of complex dynamics, providing fundamental insights into many real-world phenomena. A recent and not yet fully understood example is the collapse of coexisting synchronous and asynchronous oscillations into a globally synchronous motion found in networks of identical oscillators. Here we show that this sudden collapse is promoted by a further decrease of synchronization, rather than by critically high synchronization. This strikingly counterintuitive mechanism can be found also in nature, as we demonstrate on epileptic seizures in humans. Analyzing spatiotemporal correlation profiles derived from intracranial electroencephalographic recordings (EEG) of seizures in epilepsy patients, we found a pronounced decrease of correlation at the seizure onsets. Applying our findings in a closed-loop control scheme to models of coupled oscillators in chimera states, we succeed in both provoking and preventing outbreaks of global synchronization. Our findings not only advance the understanding of networks of coupled dynamics but can open new ways to control them, thus offering a vast range of potential new applications.

Synchrony and asynchrony coexist in a multitude of natural and man-made systems where they play key roles in their functions and dysfunctions^1^,^2^,^3^,^4^,^5^,^6^,^7^,^8^. The mechanism underlying this coexistence can be studied in models of coupled oscillator networks, simple in their structure yet complex in their dynamics. Even networks of identical coupled oscillators can segregate into two subpopulations, one with synchronous oscillations and the other with an irregular asynchronous motion^9^. Such so-called chimera states^10^,^11^ have been studied analytically^9^,^10^,^12^,^13^,^14^,^15^,^16^,^17^,^18^,^19^,^20^,^21^, numerically^9^,^10^,^12^,^13^,^14^,^15^,^16^,^17^,^18^,^19^,^20^,^21^,^22^,^23^,^24^,^25^,^26^,^27^,^28^,^29^,^30^ and experimentally^13^,^24^,^25^,^26^,^27^,^28^. While chimera states are stable in the thermodynamic limit of infinitely many oscillators^11^,^18^, a recent study has shown that for networks of finite size they can collapse into a fully synchronized state^23^. The exact mechanisms that trigger this seemingly sudden collapse, however, remain unknown.

To address this open problem, we studied the dynamics of a ring of identical phase oscillators with nonlocal coupling. Mutually close oscillators are connected by a high coupling, and with increasing distance between oscillators, the coupling strength decreases to zero. The dynamics of such networks depends in a nontrivial way on its parameters^11^, such as the number of oscillators and the range of the coupling. We fixed the parameters such that for most random initial conditions, the network entered into a chimera state and the mean lifetime prior to the chimera collapse was comparable to the maximal lifetimes reported in previous studies^15^,^23^,^24^,^25^. We here use a data-driven approach to study the dynamics of the network model. In particular, we use signal analysis to evaluate the instantaneous coherence and temporal evolution of the oscillator phases. Apart from the ring topology, no further knowledge about the network structure is used.

## Results

### The network model

Our model consists of *N* = 51 identical nonlocally coupled oscillators assumed to be placed equidistantly on a ring with circumference 51. Accordingly, all indices, sums and differences of indices are understood as modulo *N*. For *j* = 1, …, 51, the phases of the oscillators are governed by:


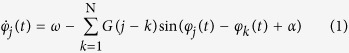


Here *ω* is the natural frequency which is set to zero, and *α* is the phase lag parameter which is set to 1.46 rad (see^22^,^23^). The coupling kernel function *G* is described in the Methods section.

### Formation and collapse of chimera states

**Figure 1:**
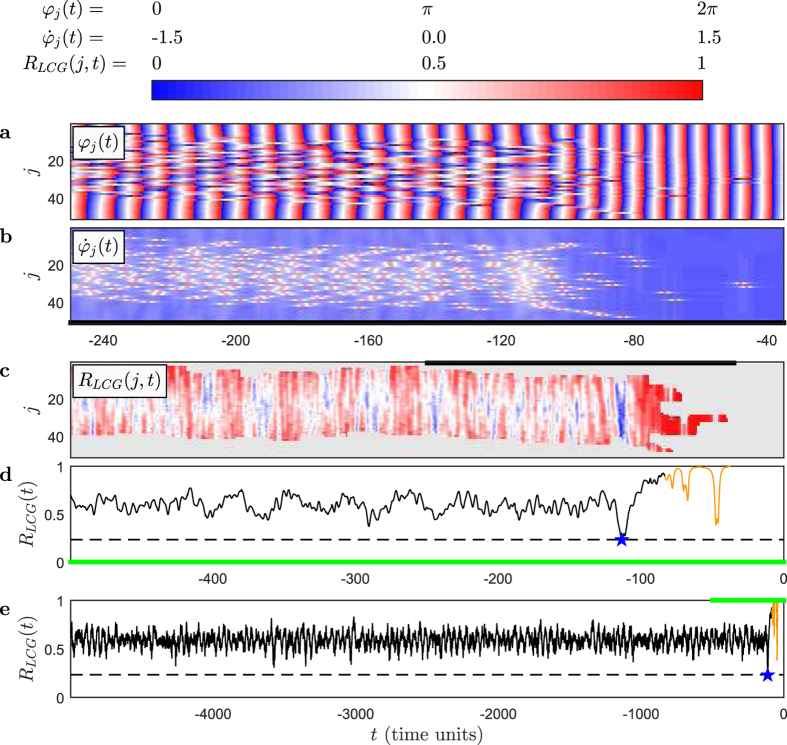
Chimera state collapses are typically preceded by hypo-coherence events. Temporal evolution of phases *φ*_*j*_(*t*) (**a**) and phase velocities 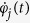 (**b**) of the network oscillators indexed by *j* show the dynamics of a chimera state collapse. Time *t* is relative to the onset of full synchronization at *t*_FS_ = 0. This chimera state collapse took place more than a million HCG oscillation periods after the formation of the chimera state. Accordingly, this figure displays only the very end of the chimera state. Due to the ring topology, oscillators at the upper and lower boundaries are neighbors. We use different time scales to optimally visualize the different quantities. Black and green abscissa segments indicate the correspondence of intervals between panels. LCG order parameter *R*_LCG_( *j, t*) (**c**) and its spatial average *R*_LCG_( *t*) on a short (**d**) and long timescale (**e**) show the hypo-coherence event at *t* = −113 time units marked by the star and dashed line. *R*_LCG_( *t*) is plotted in orange for times at which the LCG has less than 21 oscillators. Gray areas in (**c**) correspond to HCG oscillators.

Figure 1 and Supplementary Fig. 1 show different stages of the network dynamics obtained from numerical integration of the network model (see Methods). Shortly after the network is initialized with uniform random phases the chimera state is established. The network is divided into two distinct groups, i.e. the spatial symmetry of the network structure does not result in a spatial symmetry in its dynamics’ states. Instead this symmetry is broken in the way described in the following. In the *high-coherence group* (HCG), all nodes are locked to a narrow range of phases and jointly oscillate at an almost constant phase velocity. The nodes of the complementary group, in contrast, are dispersed and behave in an irregular way, including intermittent changes of the sense of rotation^23^. This second group is commonly referred to as incoherent. However, we here refer to it as *low-coherence group* (LCG) since its local order parameter (see Methods) reveals a low but non-negligible coherence (see also^26^). Oscillators at the borders between the HCG and LCG frequently switch sides. They lock onto the HCG or get disconnected from it. Therefore, the HCG group slowly drifts across the network^22^. More than a million HCG oscillation periods past its formation, the chimera state shown in Fig. 1 suddenly collapses, and the network switches to the fully synchronized state^23^. After the last irregularities have faded out, the phases of all nodes become completely locked and oscillate at constant phase velocity. The translational symmetry of the network structure is eventually also reflected by the dynamics’ states.

### Hypo-coherence events

**Figure 2:**
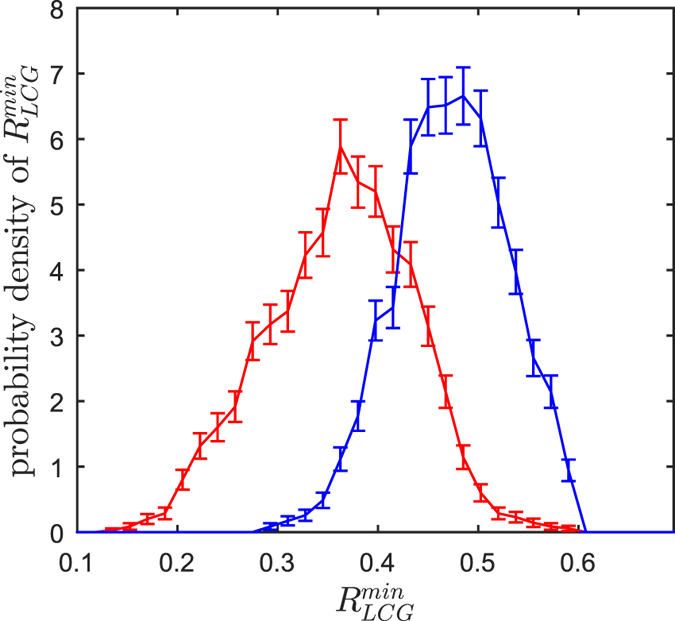
Hypo-coherence events prior to chimera collapses are significantly more prominent than controls. Probability density distribution of the depth of the last prominent minimum 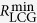 of *R*_LCG_(*t*). Distributions were estimated from 2,000 independent realizations of the network prior to the chimera collapse (red) and prior to times without collapse as control (blue). The distributions are significantly different (Wilcoxon rank sum test score: *Z* = 42.42; *p* ≪ 10^−15^). Error bars show ± one square root of the count underlying the histograms used to estimate the probability densities.

Sieber *et al*.^15^ conjectured that the seemingly sudden collapse of the chimera state occurs when finite-size fluctuations allow the network dynamics to pass through the barrier of an unstable chimera state solution to the fully synchronized stable state (see also^29^). It has not been studied so far, however, whether a specific network state facilitates this process. A priori, one might expect that the finite-size fluctuations lead to a critical number of HCG nodes or some critically high LCG coherence. Such a critical mass might initiate positive feedback, triggering a transition to the fully synchronized state. This notion is further supported by the unstable chimera state solution residing at high values of the global order parameter^15^. However, our network shows no evidence for such critical-mass mechanisms. Instead we find that the chimera collapse is typically preceded by a *hypo-coherence event* in the LCG. This seemingly counterintuitive mechanism is reflected in a prominent drop in the spatiotemporal profile of the LCG order parameter (Fig. 1c–e). Only subsequent to this hypo-coherence event do positive feedback mechanisms take over. This positive feedback, in the sense that a high synchronization leads to a further increase of synchronization, then drives the network to the fully synchronized state. We quantify these hypo-coherence events by the last prominent minimum of the LCG order parameter prior to the collapse (Fig. 1d,e). Last prominent minima determined in the same way but not followed by any collapse serve as controls. Across independent realizations of the network, minima preceding collapses are significantly lower than the controls (Fig. 2).

### Different pathways to full synchronization and their lifetimes

**Figure 3:**
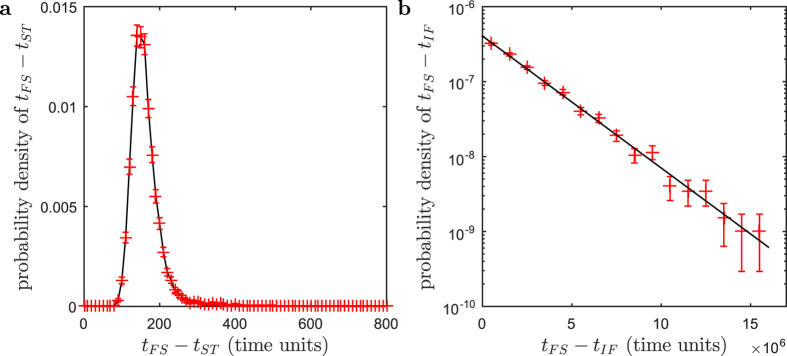
The mean lifetime of chimera states is orders of magnitudes longer than the one of immediate decays. (**a**) Probability density distribution of lifetimes in the immediate decay pathway estimated from 4,848 realizations. Here the lifetime is defined as the time between the initialization of the network with random phases at *t*_ST_ and the onset of the fully synchronized state at *t*_FS_. The black line connects data points to guide the eye. (**b**) Probability density distribution of chimera state lifetimes estimated from 2,000 independent realizations. Here the lifetime is defined as the time between the formation *t*_IF_ of the chimera state and the collapse to the fully synchronized state at *t*_FS_. The longest lifetime we observed across all 2,000 independent realizations of the network was 1.7 ⋅ 10^7^ time units. The black line corresponds to an exponential distribution with a mean of 2.5 ⋅ 10^6^ time units. Given these long lifetimes the computational load to follow all 95,152 realizations that entered into the chimera pathway was prohibitive, even using massive distributed long-term computing. In both panels error bars show ± one square root of the count underlying the histograms from which we estimated the probability densities.

How does a drop in the already low coherence of the LCG promote the onset of the fully synchronized state? When the network is initiated with random phases, it will always end up in the fully synchronized state. There are, however, two distinct pathways to this final stable state. So far we have treated the chimera pathway. In a second pathway the network synchronizes directly, without ever forming a chimera state (Supplementary Fig. S2). For our network this immediate-collapse pathway was observed in 4,848 of 100,000 independent realizations (~5%). For this pathway the distribution of lifetimes, i.e., the times from the initiation to the fully synchronized state, is right-skewed and has a mean of 162 ± 2.33 time units (mean ± standard error of mean). The maximum observed lifetime in the immediate-collapse pathway was 693 time units (Fig. 3). Out of the remaining 95,152 realizations that formed a chimera state (~95%), we continued to integrate a randomly selected subsample of 2,000 realizations until they collapsed. The resulting lifetime distribution is exponential^23^,^25^,^28^ (Fig. 3), implying that the collapse is a Poisson process^25^. Accordingly, the instantaneous probability that a collapse occurs is time-independent, the chimera state is not ageing. Using the empirical distribution mean of (2.5 ± 0.05) ⋅ 10^6^ time units, we can thus determine the probability that a chimera collapses within 693 time units, i.e. within the longest lifetime observed in the immediate-collapse pathway. It is as small as 2.8 ⋅ 10^−4^.

### Hypo-coherence events trigger the collapse of chimera states

Hence, for our network the transition rate from chimera states to the fully synchronized state is orders of magnitude lower than the one from the initial random, fully incoherent state. At first sight, this might again seem counterintuitive since for the chimera state the HCG nodes are already oscillating almost synchronously. Therefore, a first step towards full synchronization is already taken, and only the LCG nodes remain to be recruited. We conjecture that the low coherence of the LCG is non-negligible in the sense that it is protective against this recruitment. As long as the LCG has its own mean field, it is less vulnerable to be absorbed by the HCG. Once this opposition is weakened during a hypo-coherence event, the HCG can impose its rhythm and rapidly recruit all oscillators in the network. This has to be understood in a probabilistic sense. Hypo-coherence events are not fully sensitive and specific for the chimera collapse (distributions in Fig. 2 overlap), but they do render the network susceptible to suffer an immediate chimera collapse.

### Closed-loop control schemes

Results of two different closed-loop feedback schemes corroborate this interpretation. In these simulations, based on 500 independent realizations each, we transiently changed the phase lag coupling parameter *α* (see also^15^,^21^,^29^) whenever the LCG order parameter entered the range found for hypo-coherence events. Importantly, we changed *α* exclusively in couplings between LCG oscillators. In the first scheme we set *α* so as to cause a further decrease of the LCG coherence. This decreased the mean chimera lifetime from (2.5 ± 0.05) ⋅ 10^6^ to (2.6 ± 0.1) ⋅ 10^5^ time units. In the second scheme, an *α* counteracting the hypo-coherence event increased the lifetime to (7.4 ± 0.3) ⋅ 10^6^ time units. As controls we used two corresponding open-loop feedback schemes. Here changes of *α* were not triggered by hypo-coherence events but were applied periodically, matching in both frequency and duration the respective closed-loop feedback scheme. The effects were much smaller with resulting mean lifetimes of (2.6 ± 0.1) ⋅ 10^6^ and (2.9 ± 0.1) ⋅ 10^6^ time units, respectively.

### Decrease of correlation at the onsets of epileptic seizures in humans

**Figure 4:**
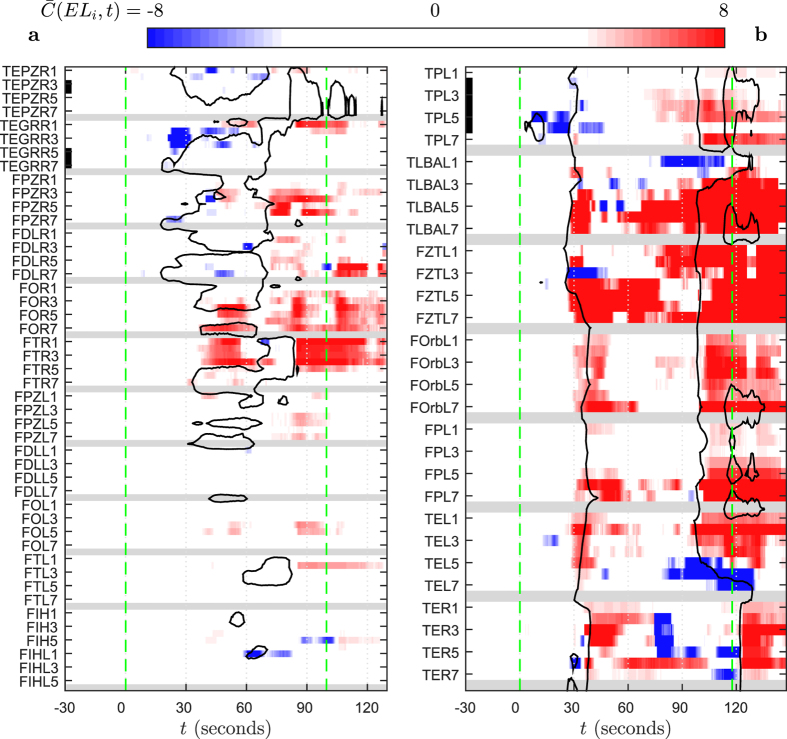
Localized decrease of correlation at the onset of epileptic seizures. Color-coded normalized slope cross correlation 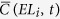 for epileptic seizures of patient A (**a**) and B (**b**). Gray is used for the last contacts on individual electrodes (having no next neighbor to calculate 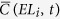 with). Ordinate labels indicate electrode names and every second recording contact number. Black bars mark electrode contacts that were located in brain tissue subsequently resected in epilepsy surgery. Green lines mark the visually detected electroencephalographic seizure onset (at *t* = 0) and offset times. Black contour lines mark the electrode-temporal range showing prominent seizure activity, assessed by thresholding the normalized absolute EEG signals slope: S(*EL*_*i*_, *t*) > 7.

Do chimera states bear any relevance for real-world phenomena? We use an example from neurology to show that phenomena analogous to hypo-coherence events preceding chimera state collapses indeed exist. Analyzing spatiotemporal correlation profiles derived from intracranial EEG recordings of seizures, we found a pronounced decrease of correlation at the seizure onsets (Early localized blue areas in Fig. 4). This decrease is most prominent for signals recorded either from within, or close to, brain regions that show the first electrographical signs of seizure activity. The subsequent spread of the seizure activity to extended brain regions is accompanied by a global increase in correlation which outlasts the actual seizure (Extended red areas in Fig. 4). We found similar patterns across further seizures from the patients shown in Fig. 4 and analogous patterns in seizures from other patients. Nevertheless, our intention is not to suggest that transient correlation decreases at seizure onsets can be found for all types of seizures in all patients or that they can be used for a reliable early detection or even prediction of epileptic seizures. To stress this point^31^, we show a sample seizure onset not exhibiting any correlation decrease in Supplementary Fig. S5.

## Discussion

The key analogy across our findings is that an outbreak of global synchronization is preceded by a transient decrease of synchronization in a subpopulation of the network. We anticipate that this represents only a first link between chimera collapses and epileptic seizures. Functions of the brain are complex and diverse and so are its dysfunctions, several of which can manifest themselves in epileptic seizures. Likewise the dynamics of coupled oscillator networks are very rich. Chimera states and chimera-like phenomena arise in a multitude of coupled systems^11^. However, they exist only for limited ranges of the network parameters^11^. Within these ranges their characteristics, stability, and mean lifetimes^23^,^24^ are influenced by the network topography, type of oscillator, and features of the coupling. In a pre-analysis, we found hypo-coherence events for extended network parameter ranges within the limits for which chimera states exist. As stated above, we fixed these parameters such that for most random initial conditions, the network entered into a chimera state and the mean chimera state lifetime was comparable to the maximal lifetimes reported in previous studies^15^,^23^,^24^,^25^. None of the network parameters were optimized with regard to the results shown herein. By narrowing the kernel and thus approaching a kernel broadness for which chimera states do not exist, we found chimera states of significantly shorter lifetimes that resemble so-called breathing chimeras^12^,^14^, characterized by a waxing and waning of the LCG (Supplementary Figs 3 and 4). Here the collapse occurs in a particularly deep breathing cycle. Accordingly, apart from hypo-coherence events, further intriguing mechanisms can underlie the collapse of chimera states. As a further example, consider a system of globally coupled oscillators with internal delayed feedback which was recently reported to show chimera-like states^32^. In contrast to our network, the fully synchronized state of this network incorporating delays is not stable. Is the healthy brain using similar mechanism not to have seizures? Along these lines, instead of contradicting our findings, the counterexamples provided in Supplementary Figs S4–S5 are promising candidates for which one can seek real-world and model counterparts, respectively.

It is important to emphasize at which conceptual level we establish a link between chimera collapses and epileptic seizures. Certainly a ring of nonlocally coupled identical phase oscillators is not a realistic model of the brain. Furthermore, our analysis cannot provide evidence that the chimera collapses and epileptic seizures are caused by the same dynamical scenario such as specific types of bifurcations. What we do show is that from the perspective of a data-driven approach both systems exhibit an analogous phenomenon, namely a transient decrease of synchronization in a subpopulation of the network which precedes an outbreak of global synchronization.

While epileptic seizures are classically considered to reflect abnormal excessive or synchronous neuronal activity^33^, our findings support the emerging view that apart from synchronization also de-synchronization is important for seizures dynamics^34^,^35^,^36^,^37^,^38^. Furthermore, there is growing evidence that brain regions showing the first electrographical signs of seizure activity at seizure onset are characterized by an elevated level of neuronal synchronization during the seizure-free interval (see^4^,^39^ and references therein). Given this prominent role that alterations of neuronal synchronization play in epilepsy, changes in synchronization are regarded as a promising feature for seizure prediction. Mormann and coworkers^40^ used the mean phase coherence^41^ as an estimate for neuronal synchronization to analyze intracranial EEG recordings from epilepsy patients. They found that the large majority of the investigated seizures were preceded by a decrease of synchronization lasting from several minutes up to a few hours. These decreases were not confined to the seizure onset zone but often involved more distant, even contralateral, areas of the brain but were not found in recordings from the seizure-free interval. Feldt and coworkers^42^ used a straightforward model of two interacting populations of integrate-and-fire neurons to explain these results of Mormann *et al*.^40^. Subsequent studies investigated whether spatiotemporal changes of neuronal synchronization, as estimated by the mean phase coherence or other quantitative EEG analysis measures, can be used to predict epileptic seizures (see^43^,^44^,^45^,^46^,^47^ for recent examples). It remains subject of an ongoing debate whether the sensitivity and specificity of current quantitative EEG analysis based seizure prediction approaches is sufficient for implementation in therapeutic devices (see^48^). On the other hand, their sensitivity and specificity are often reported to be higher than the values expected under the null hypothesis that the predictions are raised at random by a Poisson process (see^49^). However, due to the temporal correlations of the quantitative EEG analysis measure profiles from which the predictions are extracted, the memoryless Poisson process might not provide a plausible null hypothesis^31^. We emphasize that our study of EEG recordings does not aim at the prediction of epileptic seizures. We only report transient correlation decreases at seizure onsets, not prior to seizure onsets. The analogy of these transient correlation decreases at seizure onsets with hypo-coherence events prior to chimera collapse can, however, contribute to the understanding of the mechanisms underlying seizure onsets.

Results of our closed-loop feedback scheme add to previous work on the control of chimera states^15^,^20^,^21^,^29^,^30^. Sieber and colleagues^15^ introduced a time-dependence into the phase lag parameter as a function of the global order parameter. This closed-loop control scheme allowed stabilizing chimera states and suppressing the chimera collapse. Wolfrum and colleagues^29^ further studied the dynamical phenomena in this feedback system with particular emphasis on networks of a small number of oscillators. Bick and Martens^20^ implemented a closed-loop between the gradient of a local order parameter and an asymmetry in the coupling kernel function to suppress the drifting motion of the chimeras (see^22^). This allowed the authors to fix the position of chimera states at any desired target position on the ring. Isele *et al*.^30^ achieved to stabilize chimera states and to control their position by including excitable units in a network of otherwise oscillatory nodes. Omelchenko *et al*.^21^ stabilized chimera states and fixed their position in particularly small networks. For this purpose they introduced an oscillator-position dependence into the phase lag parameter as a function of the sum and difference of two local order parameters.

Our control scheme was restricted to the oscillators of the LCG. Whenever the LCG order parameter fell below a threshold, reflecting an imminent hypo-coherence event, we changed the phase lag parameter exclusively for couplings between LCG oscillators. Adjusting this intra LCG phase lag parameter to either counteract or enhance the hypo-coherence event allowed us to, respectively, suppress or promote the collapse of the chimera state. In other words, counteracting or enhancing a loss of coherence in the already weakly coherent subpopulation of oscillators can, respectively, suppress or promote the outbreak of synchronization across all oscillators. On the one hand, this supports our conclusion that the chimera state collapse is indeed triggered by hypo-coherence events. On the other hand, this is in keeping with the hypothesis that methods directed at increasing synchronization might help to control epileptic seizures^34^,^35^. To exploit the principles of our closed-loop schemes to control epileptic seizures will require identifying and precisely targeting the brain regions corresponding to the low-coherence group, for example by intracranial focal electrical neurostimulation^50^. Notwithstanding these challenges, the prospect of control can have far-reaching implications, beyond epileptic seizures. In the brain, a balanced coexistence of synchrony and asynchrony plays key roles in cognitive functions^2^. A disturbance of this balance can manifest itself not only in epilepsy^4^ but in further neurological disorders such as Parkinson’s disease^5^ or schizophrenia^3^.

## Methods

### The coupling kernel

We constructed the coupling kernel *G*( *j* − *k*) by placing a Tukey window of width *B* = 44.8 and parameter *r* = 0.45 centered at | *j* − *k*| = 0. The remaining part (23 ≤ | *j* − *k*| ≤ 25) was padded with zeros. Then, *G*( *j* − *k*) was normalized to have integral of one along the ring of 51 oscillators. Accordingly, the kernel has a constant value of 0.0228 in the center, is zero at both tails, and decays like a cosine kernel in between (Supplementary Fig. S3). The parameters *B* and *r* allow adjusting the broadness and steepness of the kernel. The higher *B*, the narrower the tails of value zero. The higher *r*, the broader the cosine part and the narrower the center of constant value. The values of *B* = 44.8 and *r* = 0.45 were used throughout this study. Exclusively for the simulation shown in Supplementary Fig. S4, we set *B* = 39.6 and *r* = 0.45.

### Choice of evaluation parameters

The procedures described below require determining several thresholds, time constants, minimal number of oscillators, etc. All values were set in an empirical manner. We adapted them to our particular network taking into account its size, typical time scales and dynamics by exploring in detail the influence of the parameters based on a small number of exemplary realizations. Importantly, our results do not depend in a sensitive way on any of these evaluation parameters. In particular, none of the values were optimized with regard to the results presented here.

### Numerical integration and initial conditions

We integrated equation (1) using a Runge-Kutta scheme of order four with fixed step size of *dt* = 0.01 dimensionless time units. Using this integration step, one obtains on the order of 800 samples per oscillation period of the coherent oscillators during the existence of the chimera state. Hence, we have a sufficiently small integration step size with regard to the dominant time scale of the dynamics. As start time we defined *t*_ST_ = 0 time units. As initial conditions we used random phases *φ*_*j*_(*t*_ST_) that were uniformly distributed between [0, 2*π*] for *j* = 1, …, *N*.

### Order parameters

To monitor the state of the network we represent the phases in the complex domain and use the length of the complex Kuramoto order parameter:


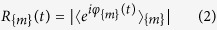


where *i* is the imaginary unit. The angular brackets < > indicate the mean value in the complex plane taken across the set of indices given in curly brackets { }. The bars | ⋅ | indicate the absolute value, which here corresponds to the Euclidean norm of the complex number to which it is applied. This order parameter assesses the instantaneous coherence of the subpopulation {*m*} of phase oscillators at time *t*. The subpopulations were adapted to the problem at hand, resulting in three different variants of the order parameter. The first variant is the global order parameter across all phases, which we use to detect the initial formation of the chimera state and the onset of the fully synchronized state:


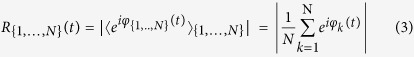


(We here write out the mean only to further illustrate the definition of Equation (2).)

The time of the initial formation of the chimera state, denoted by *t*_IF_, was defined by the smallest time *t* for which *R*_{1,…,*N*}_(*t*) > 0.7. This threshold is close to the mean of *R*_{1,…,*N*}_(*t*) during the existence of the chimera state. The time of the onset of the fully synchronized state, *t*_FS_, was defined by the smallest time *t* for which *R*_{1,…,*N*}_(*t*) > 0.99999 was found. We convinced ourselves that after crossing this threshold, the value of *R*_{1,…,*N*}_(*t*) always converged to one, hence this threshold is beyond the point of no return to full synchronization.

### Definition of high-coherence and low-coherence group

We use pair-wise order parameters to define the two complementary groups of oscillators, namely the high-coherence group (HCG) and the low-coherence group (LCG). To be included in the HCG, oscillators must belong to at least one group of at least five neighboring oscillators for which all pair-wise order parameters exceed a pre-defined threshold: If *R*_{*j*+2,*j*+1}_ > 0.995 and *R*_{*j*+1,*j*}_ > 0.995 and *R*_{*j*,*j*−1}_ > 0.995 and *R*_{*j*−1,*j*−2}_ > 0.995, then the oscillators with indices 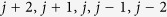 are included in the HCG. This criterion is tested for *j* = 1, …, *N*. Accordingly, the tested groups overlap, and oscillators can qualify up to five times to be included in the HCG. The number of times an oscillator qualifies is not taken into account. All oscillators that never qualify for any *j* are included in the LCG. We found that the threshold value 0.995 and the group size of five is necessary and sufficient to avoid that islands in the middle of the LCG are erroneously defined as members of the HCG. By construction, both groups cannot intersect and their union fills the entire ring of all *N* = 51 oscillators. Typically, during the existence of the chimera state, the ring of oscillators was split in one non-fragmented HCG and the complementary non-fragmented LCG. Only during the initial formation and collapse of the chimera state a fragmentation of these two groups was typically found (see Fig. 1 and Supplementary Fig. S4).

### LCG order parameter

To assess the spatial-temporal coherence profile of the LCG (see also^10^,^32^), we adapted the order parameter to be restricted to the 20 nearest neighbors of an individual oscillator *j* in the LCG: *R*_LCG_(*j, t*) = *R*_{*m*}_(*t*) for 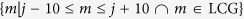. To further condense this information, we took the average 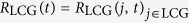. Here the angular brackets < > indicate the mean value in the real-valued domain of the order parameter.

### Last pronounced minima, originals and controls

To quantify the hypo-coherence events, we determined the last prominent minimum of the LCG order parameter *R*_LCG_(*t*) prior to the onset of full synchronization. We denote this last prominent minimum by 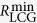. To determine it, we at first estimated the median of *R*_LCG_(*t*) across the chimera lifetime. We then went to the last time instant *t*_ON_ prior to the onset time of the fully synchronized state *t*_FS_ at which *R*_LCG_(*t*) crossed its median from above and the number of oscillators in the LCG was at least 21. This median value was used to initialize 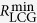. The jump backwards from *t*_FS_ to *t*_ON_ is necessary in order not to get trapped in the prominent fluctuations of *R*_*LCG*_(*t*) observed during the acute break-down of the LCG (see right end of Fig. 1d,e). From *t*_ON_, we went gradually backward in time. Whenever the value of *R*_*LCG*_(*t*) was smaller than the current value of 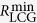, this value was stored as new value of 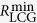. This process was stopped as soon as no new minimum was found within 10 time units after the last update of 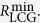 This value of 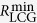 yields the last prominent minimum of the LCG order parameter *R*_LCG_(*t*).

To estimate the null distribution of 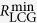, we repeated the exact same process using as initial times 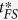 random time instants during the lifetime of chimera states at which no chimera collapse and no onset of full synchronization took place for the next 2,500 time units.

### Detection of immediate decay

After the initial formation of the chimera state, the HCG and the LCG coexist (see Supplementary Fig. S1). This formation process can be traced in the global order parameter *R*_{1,…,*N*}_(*t*). It rises quickly, crosses the threshold used to define the initial formation time (*R*_{1,…,*N*}_(*t*) > 0.7, see above), and then fluctuates around a stable mean value of *R*_{1,…,*N*}_(*t*) ≈ 0.7. In contrast, in the immediate decay pathway (see Supplementary Fig. S2), a chimera state is never formed. This immediate decay can be detected readily in *R*_{1,…,*N*}_(*t*). It rises quickly, crosses the threshold of *R*_{1,…,*N*}_(*t*) > 0.7, but never stabilizes around a mean value. Rather it directly approaches and crosses the threshold of full synchronization (*R*_{1,…,*N*}_(*t*) > 0.99999, see above). Accordingly, an immediate decay was detected whenever *R*_{1,…,*N*}_(*t*) went directly from *R*_{1,…,*N*}_(*t*) > 0.7 to *R*_{1,…,*N*}_(*t*) > 0.99999. Conversely, we discarded an immediate decay if at any time after *t*_IF_ we found *R*_{1,…,*N*}_(*t* + 40) < 0.9*R*_{1,…,*N*}_(*t*). This latter exclusion criterion is fulfilled shortly after *R*_{1,…,*N*}_(*t*) starts to fluctuate around its mean, i.e., once the chimera state is consolidated.

### Definition of lifetimes

The lifetime in the immediate decay pathway is defined as the length of the time interval between the initialization of the network with random phases and the onset of the fully synchronized state: *t*_FS_ − *t*_ST_ = *t*_FS_. The lifetime of the chimera state is defined as the length of the time interval between the formation of the chimera state and the onset of the fully synchronized state: *t*_FS_ − *t*_IF_. For practical purposes, this coincides with the length of the time interval between the initialization of the network with random phases and the onset of the fully synchronized state, since the lifetime of the chimera state is orders of magnitude longer than the time needed for the initial formation of this state.

### Closed- and open-loop control schemes

We used two closed-loop control schemes to either provoke or counteract collapses of chimera states. In both schemes, the control was activated whenever the LCG order parameter fell below a predefined threshold: *R*_LCG_(*t*) < 0.45, i.e. when this order parameter entered the range typically covered by hypo-coherence events (see Fig. 2). In both schemes, the control consisted of a change of the phase lag parameter *α* from its otherwise fixed value of 1.46 rad (see Equation 1). A similar control strategy was applied in refs 15, 21 and 29. In contrast to this previous work, however, we exclusively changed the phase lag parameter for couplings between pairs of oscillators both belonging to the LCG. The *α* of couplings between pairs of HCG oscillators and between LCG and HCG oscillators was never changed. In the scheme that was used to provoke chimera collapses, we increased the intra-LCG phase lag parameter to 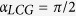 rad > 1.46 rad. The effect of this increase is a further reduction of the coherence of the LCG oscillators. In the scheme used to counteract chimera collapses, we used 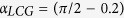 rad <1.46 rad. The effect of this decrease in turn is a recovery of the LCG oscillator coherence. In both schemes, the control was turned off five time units after its activation or once *R*_LCG_(*t*) > 0.45. No control was applied prior to the initial formation of the chimera state or when the number of LCG oscillators dropped below 21, a characteristics of an already ongoing chimera collapse (see Fig. 1).

To test whether this closed-loop control is indeed more effective when triggered by hypo-coherence events, we used two corresponding open-loop control schemes. These followed the same steps as their corresponding closed-loop counterparts. In the open-loop schemes, however, the control was activated and deactivated periodically regardless of the LCG order parameter. The inter-control-interval and duration of individual controls was adjusted to match the respective means of the corresponding closed-loop schemes.

### Electroencephalographic recordings (EEG) - Recording techniques and clinical data

Electroencephalography is a key diagnostic tool in epilepsy patients suffering from pharmacoresistent epilepsies and who are potential candidates for epilepsy surgery. When extracranial surface (“scalp”) EEG is insufficient to precisely localize the epileptogenic regions of the brain, EEG can also be recorded intracranially from preselected brain regions using implanted strip, grid and depth electrodes. We here analyze intracranial EEG recorded at the Inselspital Bern, Switzerland, from three patients before, during and after seizures.

For data acquisition AdTech electrodes (Wisconsin, USA) and a NicoletOneTM recording system (VIASYS Healthcare Inc., Wisconsin, USA) were used. Before analysis EEG signals were down-sampled to a sampling time of Δt = 1.953 ms, re-referenced against the median of all the channels free of permanent artifacts as judged by visual inspection by an experienced epileptologist (K.S.) and digitally band-pass filtered between 0.5 and 150 Hz using a fourth-order Butterworth filter. Forward and backward filtering was applied to minimize phase distortions. All EEG recordings were carried out prior to and independently from our retrospective analysis of the data. Importantly, these recordings are not experimental but were recorded for clinical diagnostics and treatment of each individual patient only. In accordance with approved guidelines, the EEG recordings and additional information (age, localization of seizure onset, etiology and postsurgical outcome) was anonymized prior to our analysis. In addition, all patients had given written informed consent that their data from long-term EEG might be used for research purposes.

Patient A is a 27 year old male patient who suffered from lateral temporal lobe epilepsy (LTLE) with seizures starting in the left hemisphere. After resection of parts of the left temporal lobe he became completely seizure free with follow up of one year. Patient B is a 49 year old female frontal lobe epilepsy patient with focal cortical dysplasia in the right middle frontal gyrus. Resection of the dysplasia led to significant reduction of the seizure rate (follow up four years). Patient C is a 20 year old male LTLE patient. Seizures started in the right anterior temporal lobe though with fast bilateral spreading. Seizure rate was significantly reduced after surgical removal of the right temporal pole (follow up three years).

### Normalized slope cross correlation from EEG recordings

We here follow Rummel and coworkers^37^ and apply the zero-lag linear cross correlation of the EEG signal slopes. We measure time *t* in seconds relative to the visually determined electroencephalographic seizure onset. Let *EL* be the name of a recording electrode carrying a total of *c* individual recording contacts. Furthermore, let *x*(*EL*_*i*_, *t*) denote the slope of the EEG signal, defined by the difference between the EEG signal amplitude measured at time *t* and *t* − Δ*t* at the *i*-th contact. Then we denote by *C*(*EL*_*i*_, *t*) the zero-lag linear cross correlation between the slope *x*(*EL*_*i*_, *t*) and the slope of the adjacent contact *x*(*EL*_*i*+1_, *t*), for 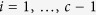. The correlation between non-neighboring channels on individual electrodes or across electrodes was not considered. *C*(*EL*_*i*_, *t*) was determined in a moving window analysis with a window length of 5 seconds and 90% overlap between subsequent windows. For each individual signal pair, *C*(*EL*_*i*_, *t*) was normalized using its mean 〈⋅〉 and standard deviation *σ*(⋅) both taken across a reference interval 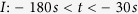:


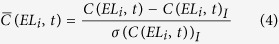


Accordingly, 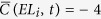 or 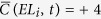 indicates that the *C*(*EL*_*i*_, *t*) is 4 standard deviations lower or higher, respectively, than during the reference interval *I*. Temporal differentiation is equivalent to multiplying the amplitude of the Fourier transform at any frequency with the value of the frequency. For typical EEG power spectra this is equivalent to whitening the signals. Thus, by using the slope of the EEG signals rather than directly the EEG signals, we focus on the correlation in the faster oscillations.

### Normalized absolute slope of EEG signals

We follow Schindler and coworkers^35^ and define seizure activity by using the normalized absolute slope of the EEG. This is defined for 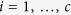 as:


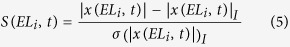


Here |⋅| denotes the absolute value. All other quantities, including the parameters of the moving window analysis, are defined as in the previous section. The normalized absolute slope of the EEG is large for both high-amplitude slow activity and low-amplitude fast activity, which are both typically observed at the onset and during intracranially recorded epileptic seizures.

## Additional Information

**How to cite this article**: Andrzejak, R. G. *et al*. All together now: Analogies between chimera state collapses and epileptic seizures. *Sci. Rep.* **6**, 23000; doi: 10.1038/srep23000 (2016).

## Supplementary Material

Supplementary Information
