# Supplementary material for: All together now: Analogies between chimera state collapses and epileptic seizures
Source: Sci Rep. 2016 Mar 9;6:23000. doi: 10.1038/srep23000 (PMC4783711; doi:10.1038/srep23000)
Supplement: Supplementary Information [file srep23000-s1.pdf]

Supplementary Information

All together now: Analogies between chimera state collapses and epileptic seizures

Ralph G. Andrzejak[1], Christian Rummel[2], Florian Mormann[3], Kaspar Schindler[4]

Supplementary Figure 1-5

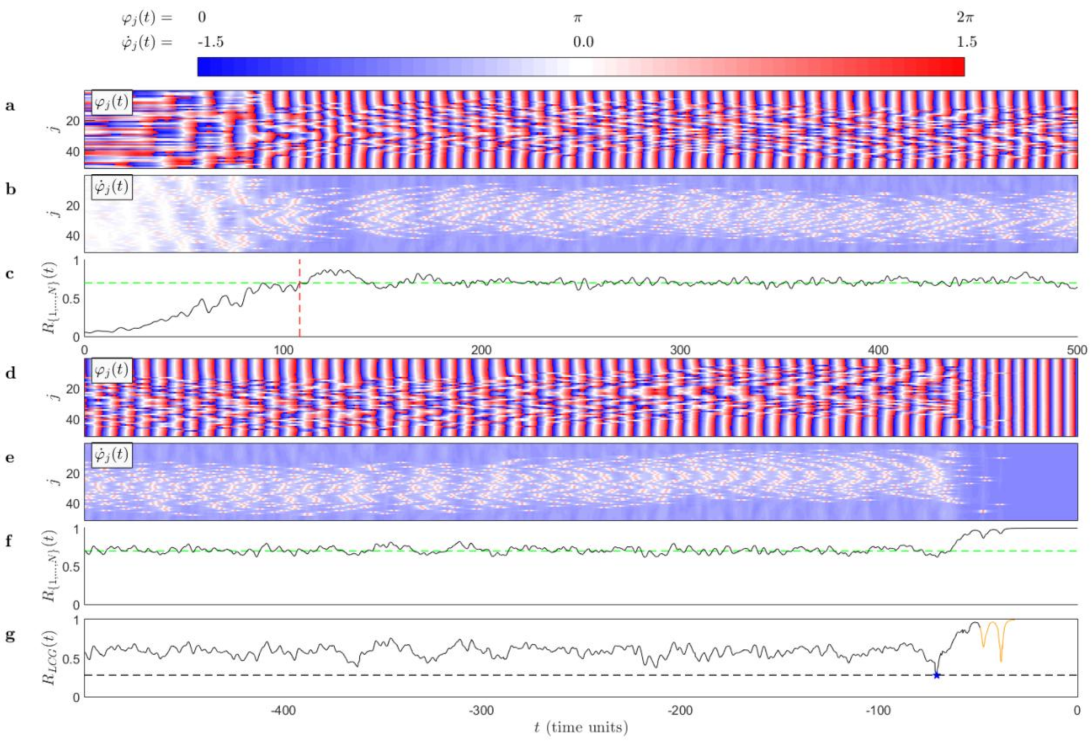

Supplementary Fig. 1: Formation and collapse of a chimera state

Panels **(a, b, d, e)** are analogous to panels (a) and (b) of Fig. 1, but for longer intervals including the formation and collapse of a chimera state. Phases $\varphi_j(t)$ (**a**) and phase velocities $\dot{\varphi}_j(t)$ (**b**) after the initialization with random phases. Time is given relative to the start of the network at t_{ST}. The global order parameter $R_{\{1,\dots,N\}}(t)$ in panel **(c)** is used to detect the time of the initial chimera state formation t_{IF} indicated by the red vertical line. The green horizontal line indicates the threshold used in this detection. During the existence of the chimera state, HCG oscillators show a regular ripple pattern in their phases and only little fluctuations in their phase velocities. Oscillators belonging to the LCG show an irregular phase evolution and time-dependent phase velocities. Red spots in the profile of the phase velocities $\dot{\varphi}_j(t)$ correspond to intermittent changes of the sense of rotation. Panels **(d-f)** are the same as **(a-c)** but here for the collapse of a chimera state. Time is given relative to onset of the fully synchronized state at t_{FS}. For this realization, the collapse took place at $0.7 \cdot 10^6$ time units. While the global order parameter can be used to determine the times t_{IF} and t_{FS}, it does not show the hypo-coherence event. This can only be detected in the LCG order parameter $R_{\mathrm{LCG}}(t)$ which is shown in panel **(g)**. We applied circular shifts to the indices of the ring to maintain the LCG in the center of the display.

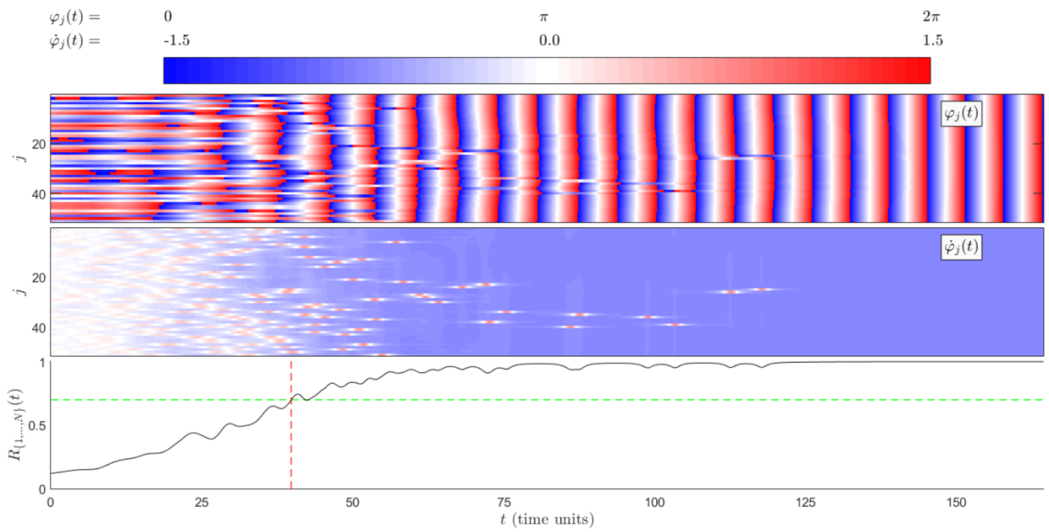

Supplementary Fig. 2: For some 5% of the initial conditions the network immediately collapsed into the fully synchronized state

These plots are analogous to panels **(a-c)** of Supplementary Fig. 1, but here no chimera state is formed after the network is initialized with random phases. $R_{\{1,...,N\}}(t)$ does not stabilize around the mean value found for chimera states (green horizontal line). Instead the network directly collapses to the fully synchronized state.

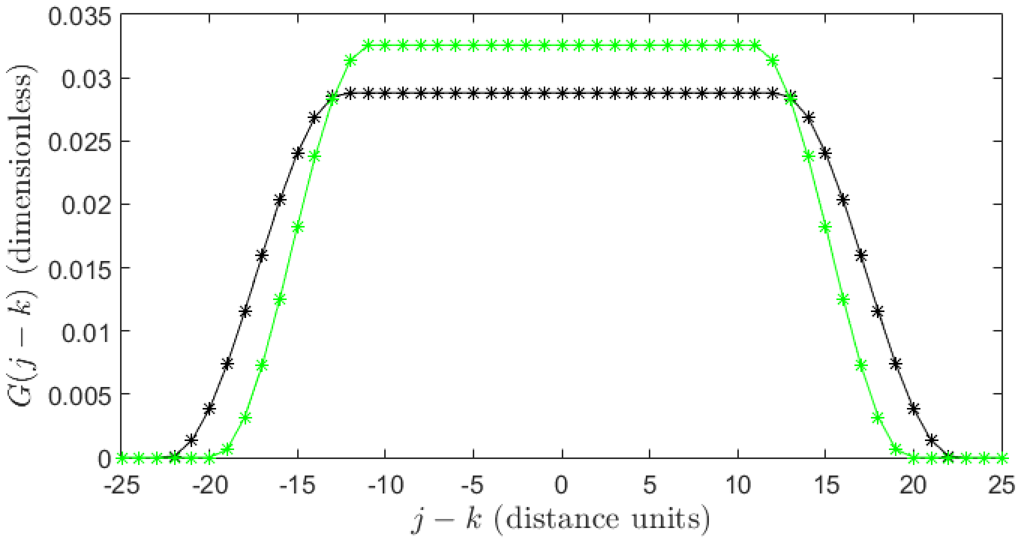

Supplementary Fig. 3: Coupling kernel functions

Nonlocal coupling kernels $G(j-k)$ as a function of the distances between oscillators on the ring $j-k$. Black: Kernel with default parameters used throughout the study ($B=44.8$ and r$=0.45$) Green: Kernel with $B=39.6$ and r$=0.45$, values exclusively used for the simulation shown in Supplementary Fig. 4. The kernels are defined for all values between -25 and 25. They are, however, only sampled at integer values, indicated by asterisks, since the distances between oscillators are integers.

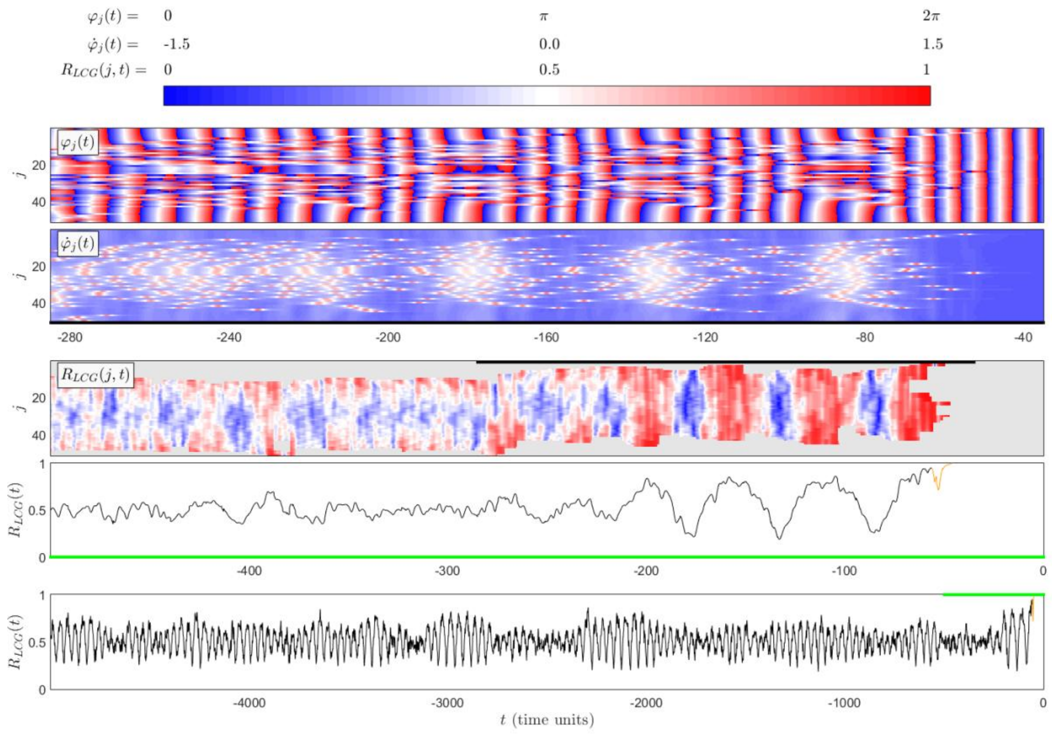

Supplementary Fig. 4: A counterexample - dynamics observed for a coupling narrower kernel

Same as Fig. 1, but using a narrower kernel (see Supplementary Fig. 3). This kernel results in a different dynamics resembling a breathing chimera state.

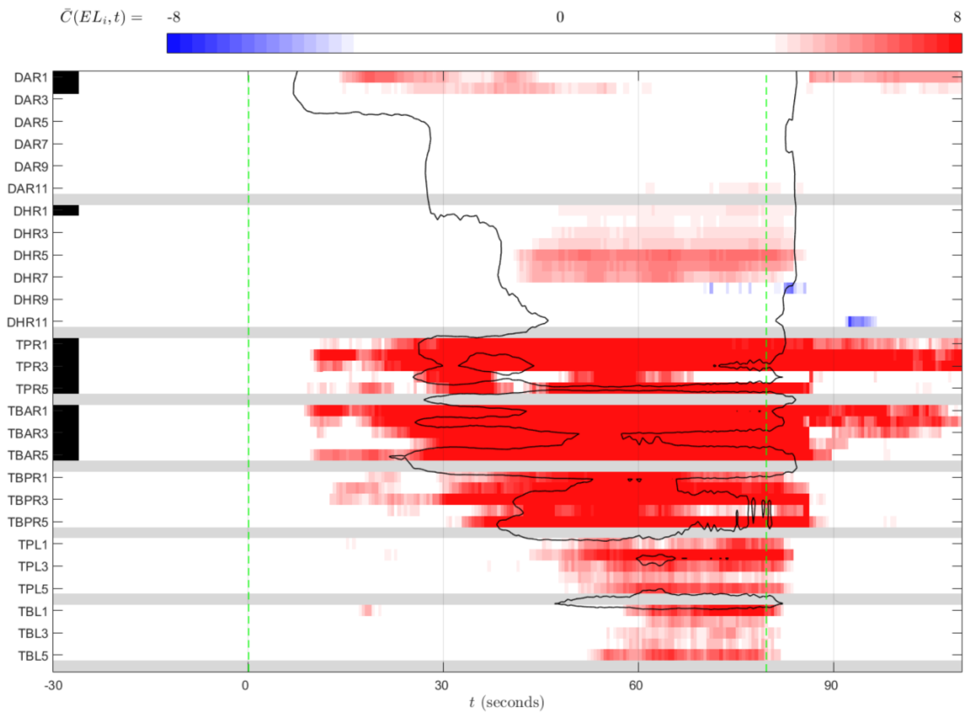

Supplementary Fig. 5: A counterexample – epileptic seizure onset without decrease of slope cross-correlation

Same as Fig. 4, but here for a seizure from patient C.
